# Silicon-Rhodamine Functionalized Evocalcet Probes Potently and Selectively Label Calcium Sensing Receptors *In Vitro*, *In Vivo*, and *Ex Vivo*

**DOI:** 10.1021/acsptsci.4c00096

**Published:** 2024-04-25

**Authors:** Daniel Bátora, Jérôme P. Fischer, Reto M. Kaderli, Máté Varga, Martin Lochner, Jürg Gertsch

**Affiliations:** † Institute of Biochemistry and Molecular Medicine, University of Bern, 3012 Bern, Switzerland; ‡ Graduate School for Cellular and Biomedical Sciences, University of Bern, 3012 Bern, Switzerland; § Department of Visceral Surgery and Medicine, Inselspital, Bern University Hospital, University of Bern, 3010 Bern, Switzerland; ∥ Department of Genetics, ELTE Eötvös Loránd University, 1117 Budapest, Hungary

**Keywords:** calcium sensing receptor, fluorescent small-molecular probe, live imaging, fluorescence-guided surgery, GPCRs

## Abstract

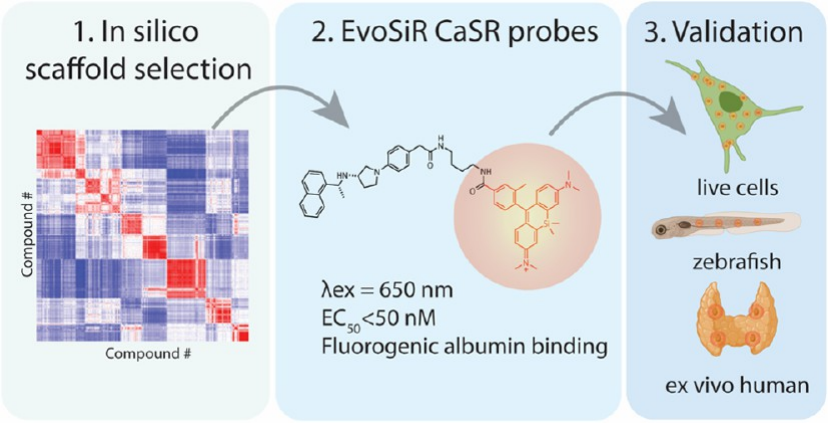

The calcium sensing receptor (CaSR) is a ubiquitously expressed G-protein coupled receptor (GPCR) that regulates extracellular calcium signals *via* the parathyroid glands. CaSR has recently also been implicated in noncalcitropic pathophysiologies like asthma, gut inflammation, and cancer. To date, molecular tools that enable the bioimaging of CaSR in tissues are lacking. Based on *in silico* analyses of available structure–activity relationship data on CaSR ligands, we designed and prepared silicon-rhodamine (SiR) conjugates of the clinically approved drug evocalcet. The new probes EvoSiR4 and EvoSiR6, with differing linker lengths at the evocalcet carboxyl end, both showed a 6-fold and 3-fold increase in potency toward CaSR (EC_50_ < 45 nM) compared to evocalcet and the evocalcet-linker conjugate, respectively, in an FLIPR-based cellular functional assay. The specificity of the EvoSiR probes toward CaSR binding and the impact of albumin was evaluated in live cell experiments. Both probes showed strong albumin binding, which facilitated the clearance of nonspecific binding interactions. Accordingly, in zebrafish embryos, EvoSiR4 specifically labeled the high CaSR expressing neuromasts of the lateral line *in vivo*. EvoSiR4 was also assessed in human parathyroid tissues *ex vivo*, showing a specific absolute CaSR-associated fluorescence compared to that of parathyroid autofluorescence. In summary, functionalization of evocalcet by SiR led to the preparation of potent and specific fluorescent CaSR probes. EvoSiR4 is a versatile small-molecular probe that can be employed in CaSR-related biomedical analyses where antibodies are not applicable.

Beyond its established role as an intracellular second messenger, calcium (Ca^2+^) is also recognized as an important extracellular signaling molecule.^1,2^ The calcium sensing receptor (CaSR) is the major class C G-protein coupled receptor (GPCR) that integrates extracellular calcium signals and controls calcium homeostasis in the parathyroid glands^3^ and kidneys^4^ by regulating parathyroid hormone production. However, the apparently ubiquitous expression of the CaSR across tissues^5^ underscores the less understood roles of CaSR signaling in a wide variety of physiological processes, for instance, its involvement in airway smooth muscle contractility,^6^ inflammation,^7^ taste modulation,^8^ and neuronal excitability.^9,10^ Furthermore, dysfunctional CaSR signaling has been implicated in noncalcitropic diseases such as asthma,^11^ colorectal inflammatory conditions such as inflammatory bowel disease (IBD)^12^ as well as Alzheimer’s^13^ and cardiovascular disease (CVD),^14^ making this GPCR an attractive potential target for the development of novel therapeutic interventions and as possible disease marker. The recent surge in research on the possible roles of CaSR and its physiological roles therefore requires the development of versatile molecular tools to visualize, monitor, and modulate CaSR. While numerous diverse ligands have been developed to modulate CaSR activity,^15^ imaging probes remain scarce. Given the limitations of using antibodies, small-molecule probes offer versatile biomolecular tools for life imaging.^16^

To date, the development of the small-molecular chemical space for CaSR binding ligands has been largely spurred by clinical needs in the therapeutic areas of hyperparathyroidism and osteoporosis.^17^ It comprises L-amino acid and peptide-based moieties that act as positive allosteric modulators (PAMs) at the extracellular Venus-flytrap (VFT) domain and arylalkylamine or quinazolinone containing PAMs and negative allosteric modulators (NAMs) that are specific to the seven transmembrane (7TM) domain of the receptor.^18^ To our knowledge, empirical data on the impact of larger fluorophore conjugates such as those found in fluorescent small-molecular conjugates (FMSCs) on CaSR binding potency and specificity are lacking.

FMSCs have been extensively used for the exploration of ligand binding sites, the quantification of ligand receptor binding interactions in competition experiments, the visualization of receptor expression in cells and tissues, and in providing a fluorescent readout of target engagement.^19^ The major objectives in the optimization of FMSCs start with the selection of the appropriate chemical scaffold to which the introduction of large fluorescent tags does not diminish protein binding affinity. Interrogation of target specificity is critical, which depends on inherent features of the chemical scaffold and the optimization of both the linker and the fluorophore. High fluorogenicity of the probe in a receptor-bound state is favorable. High albumin binding can further reduce nonspecific background signals in vascularized tissues and enhance the signal-to-noise ratio when washing steps during the labeling procedure are feasible.

In the current study, we generated the first CaSR-binding FMSCs called EvoSiR, which were designed from the molecular scaffold of evocalcet, an FDA-approved PAM calcimimetic drug.^20^ Based on structure-activity relationship (SAR) analyses, the evocalcet carboxyl group was found to be optimal for SiR functionalization, which was also confirmed by two recent Cryo-EM studies.^21,22^ Using a validated FLIPR-based functional CaSR assay, spectrofluorometric analysis, as well as *in vitro*, *ex vivo*, and *in vivo* CaSR labeling studies, we demonstrate that EvoSiR is potent and specific enough to be applied for live imaging, even without extensive washing with albumin.

## Materials and Methods

### Chemicals

Calcein AM (cat. no. 22003) and Fluo-8 AM (cat. no. 21080) were purchased from AAT Bioquest, aliquoted to 50 μg, and stored at −20 °C. 4-(4-Diethylaminostyryl)-1-methyl-pyridinium-iodid (DiAsp) and NPS-2143 hydrochloride were purchased from Sigma-Aldrich and kept at −20 °C at a stock concentration of 50 mM in DMSO.

### General Procedures for Chemical Synthesis

The chemical syntheses of EvoSiR probes are depicted in the Results Section. The detailed description of all synthesis procedures plus the ^1^H NMR, ^13^C NMR, and high-resolution mass spectrometry (HRMS) data are reported in the Supporting Information. The purity of the molecules was also assessed using high-performance liquid chromatography (HPLC). All reactions requiring anhydrous conditions were performed in a heat gun, oven, or flame-dried glassware under inert atmosphere (N_2_ or Ar). Silica gel 60 Å (40–63 mm) from Sigma-Aldrich was used for dry loads. Flash column chromatography was performed on a Teledyne Isco CombiFlash Rf+ with the corresponding RediSep prepacked silica cartouches unless otherwise stated. Thin layer chromatography (TLC) was performed on Machery & Nagel Alugram xtra SIL G/UV 254 visualization under ultraviolet (UV) light (254 nm) and/or (366 nm) and/or by dipping in anisaldehyde stain and subsequent heating. Commercial reagents and solvents (Acros Organics, Fluorochem, Grogg Chemie, Hänseler, Sigma-Aldrich, Lubio) were used without further purification unless otherwise stated. Dry solvents for reactions were distilled and filtered over columns of dry neutral aluminum oxide under positive argon pressure. Solvents for extraction and flash chromatography were used without prior purification.

^1^H and ^13^C NMR spectra were recorded on a Bruker AVANCE-300 or 400 spectrometer operating at 300 or 400 MHz for ^1^H and 75 or 101 MHz for ^13^C at room temperature unless otherwise stated. Chemical shifts (δ) are reported in parts per million (ppm) relative to tetramethylsilane (TMS) calibrated using residual signals of the solvent or TMS. Coupling constants (*J*) are reported in Hz. HRMS analyses and accurate mass determinations were performed on a Thermo Scientific LTQ Orbitrap XL mass spectrometer using ESI ionization and positive or negative mode. HPLCs were measured on a Thermo Scientific UltiMate 3000 HPLC with H_2_O + 0.1% TFA and MeCN + 0.1% TFA as eluents on an Acclaim 120 C18 5 μm 120 Å (4.6 × 150 mm^2^) column. The silica-rhodamine carboxylic acid (SiR-CO_2_H) and the corresponding *N*-hydroxysuccinimide ester (SiR-NHS) were prepared according to procedures previously described elsewhere.^23^

### *In Silico* Analysis of Chemical Scaffolds

Assays with measured CaSR activity (AID) of at least 20 entries were queried from PubChem. Chemical structures were represented in the PubChem two-dimensional (2D) fingerprint format, which encodes the presence or absence of chemical substructures in a compound using 881 bits.^24^ Clustering was performed using the *k*-means algorithm,^25^ and the optimal number of clusters were selected by applying the *kneedle* algorithm on the inertia scores.^26^ Within each AID, we calculated the Tanimoto similarity index for each molecule and the most potent molecule of the assay.^27^ For the quadrant analysis, a cutoff value of 0.1 μM was used for the CaSR activity concentrations (AC_50_), below which a compound was considered as a potent CaSR ligand.

### Functional CaSR Assays, Cell Culture

Hamster lung fibroblasts (CCL39, CaSR−) expressing the recombinant human CaSR (HCAR, CaSR+) and nontransected controls were kindly provided by Novartis AG and were kept in standard culturing conditions (37 °C, 5% CO_2_) in 1/2 DMEM (Roth, 9005.1)—1/2 F-12 (Sigma, N4888–500 mL) media containing 10% FBS. Media for the HCAR cells contained 500 μg/mL Geneticin as a selection antibiotic (Thermo Fisher, 10131035). Upon reaching 90–95% confluence, cells were detached and seeded into 96-well plates (PerkinElmer, 6005182) at a concentration of 35,000 cells per well and were incubated for 24 h before the experiment. For the functional CaSR calcium assay, cells were first washed twice with HBSS medium (Sigma, H6648), and were incubated in HBSS containing 5 μM Fluo-8 AM and 0.1 mM CaCl_2_ for 30 min at 37 °C. Calcium transients were measured upon addition of 0.5 mM CaCl_2_ with the FLIPR Tetra high-throughput cellular screening systems using the 470–495 nm LED excitation and 515–575 nm bandpass emission filters. Probe solutions contained 0.5% DMSO and were added to the cells 10 min prior to the measurement. d*F*/*F*_0_ values were calculated as a change in the peak fluorescence value of the transient for the first 200 data points normalized to the background fluorescence of the well. For estimating CaSR binding affinity, CCL39 and HCAR cells were seeded at a concentration of 20,000 cells per well in 96-well plates (PerkinElmer, 6005182) and were incubated for 24 h before the experiment. Both cells were washed with HBSS medium twice and then incubated for 10 min at 37 °C in HBSS medium containing 0.1% BSA in the presence or absence of 10 μM evocalcet. Upon the removal of media, cells were incubated in various concentrations of EvoSiR4 and EvoSiR6 in HBSS containing 0.1% BSA for 30 min at 37 °C and were subsequently washed 3 times with 0.1% BSA and resuspended in DMSO before the fluorometric measurement. The fluorescence was measured using a Tecan GENios pro microplate reader with the following parameters: λ_ex_ = 612 nm, λ_em_ = 670 nm, gain = 50 AU. Data for the EvoSiR probes and the evocalcet competition is reported as CaSR-specific fluorescence, which was calculated by dividing the absolute fluorescence of the HCAR cells with the absolute fluorescence of the CCL39 cells for each concentration.

The effects of EvoSiR probes and evocalcet on the viability of CCL39 and HCAR cells were evaluated using the Calcein AM assay as described by the manufacturer’s protocol (AAT Bioquest, 22002) with a working concentration of 10 μM. For the assay, cells were incubated in various concentrations of the compounds for 72 h. For positive controls, cells were incubated in culture media containing maximum 10% DMSO in a two-step dilution series for 72 h. Upon incubation in the Calcein AM solution for 1 h at 37 °C, the fluorescence readouts were retrieved using the Tecan GENios pro microplate reader (λ_ex_ = 485 nm, λ_em_ = 535 nm, gain = 25 AU)

### Photophysical Characterization of EvoSiR

The photophysical characterization was carried out using an Agilent Cary Eclipse spectrofluorometer and an Agilent Cary 60 UV–visible (UV–vis) spectrophotometer. For the calculation of the extinction coefficient (ε), absorbance was measured for the concentrations of 150–1350 nM for all compounds and the cuvette length was 1 cm. For relative quantum yield measurements (Φ), the Φ_r_ value of 0.68 for the well-characterized Rhodamine B was taken in 94% ethanol as a reference standard.^28^ For Rhodamine B, peak absorbance values at 545 nm were compared to the λ_em_ of 567 nm. For the SiR probes, peak absorbance values at 654 nm were compared to the λ_em_ of 670 nm. All measurements were carried out in 94% ethanol at 750 nM concentration of the compound. The Φ for each compound was calculated using the following equation
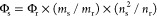
where Φ_s_ depicts the quantum yield of the sample, Φ_r_ corresponds to the quantum yield of the reference solution, *m*_s_ corresponds to the slope of the absorbance-fluorescence regression plot of the sample, *m*_r_ depicts the slope of the absorbance-fluorescence regression plot of the reference solution, and *n*_s_ and *n*_r_ correspond to the refractive indexes of the sample and reference solution solvent, respectively, which was 1.3617 for 94% ethanol. For the determination of fluorescence lifetimes (τ), the scan gate time was 0.005 ms, the delay time was 0 ms, the number of flashes was 10, the total decay time was 1 ms, and the decay curves were determined by averaging 20 cycles.

### Spectrofluorometric Analysis of Fluorogenicity and Quenching

Spectrofluorometric measurements were carried out by using the Agilent Cary Eclipse instrument. The voltage of the photomultiplier tube (PMT) was set to 600 for EvoSiR4 and EvoSiR6, and for 500 for SiR (**4**). Fluorescence of the probes was measured at concentrations ranging from 30 to 3000 nM in PBS containing various amounts of bovine serum albumin (BSA) (Sigma, A7906). Quenching experiments for the calculation of BSA binding were performed by adding a 1:1 molar ratio of tannic acid dissolved in _dd_H_2_O (Sigma, 403040) to the solution prior to measuring fluorescence.

### *In Vitro* Labeling of Stably Transfected CaSR Expressing and Nontransfected Control Cells

HCAR (CaSR+) and CCL39 (CaSR−) cells were kindly obtained from Novartis AG (Basel, Switzerland) and validated in house. The cells were seeded into μ-slide 18 well plates (ibidi, 81816) at a density of 5000 cells per well and were incubated for 24 h under standard culturing conditions. As our preliminary data indicated considerable nonspecific labeling of dying cells and cellular debris, a viability staining was introduced to ensure probe specificity.^29^ Thus, after washing twice with HBSS, cells were incubated in 10 μM Calcein AM solution containing 0.02% Pluronic F-127 and 0.1% DMSO for 45 min at 37 °C. The solution was then removed and washed twice in HBSS before the addition of the HBSS solution containing the SiR probes. All probe solutions were protected from light exposure and contained 0.5% DMSO and various concentrations of BSA depending on the experiment. Incubation with the fluorescent probes was 20 min long, after which a single washing step was introduced with HBSS before imaging. Images of the cells (Calcein: FITC channel, SiR: Cy5 channel) were acquired with a Nikon Eclipse TI2 inverted wide-field fluorescence microscope using a 60× magnification oil immersion objective (Nikon CFI Plan Fluor, 60×, NA = 0.85). For each well, a minimum of 3 images were acquired. For each image, cells were segmented to generate labels on the FITC channel postacquisition using the deep neural network-based Cellpose package in Python 3.9 (model_type: cyto2, diameter: 200, flow_threshold: 0.4).^30^ To normalize pixel intensity values across images, for each image, a background value was calculated by taking the average pixel intensity value of the pixels in which no cellular label was present and were negative for the EvoSiR label (triangle thresholding used). For each cell, the skewness of the value distribution and the mean of the Cy5 pixel intensities were calculated. The procedure for evaluating the long-term labeling (72 h) of EvoSiR probes on CCL39 and HCAR cells was similar, with the following modifications: images were retrieved using 10× magnification air objectives (Nikon CFI Plan Fluor 10×, NA = 0.3), in the neural network model, the diameter parameter was set to 30, and only the mean Cy5 pixel intensities per cell object were retrieved.

### Zebrafish Husbandry

Transgenic *Tol056*(31) and *Tg(7xTCF-Xla.Siam:GFP)*(32) zebrafish (*Danio rerio*) used in this study were maintained and bred within the fish facility of ELTE Eötvös Loránd University (Budapest, Hungary) according to standard protocols.^33,34^ The protocols used in this study were approved by the Hungarian National Food Chain Safety Office (Permit Number: XIV-I-001/515–4/2012).

### *In Vivo* Labeling and Imaging of Zebrafish Embryos

For the neuromast labeling experiments, wild-type 4–6 days post fertilization (dpf) zebrafish larvae were incubated in 200 μL of E3 medium containing different doses of the SiR probes (500–5000 nM), 2.5 μM 4-(4-diethylaminostyryl)-1-methyl-pyridinium-iodid (DiAsp, Sigma, D3418), which specifically labels hair cells in the lateral line in 0.5% DMSO for 30 min (35). Next, washing and anesthesia were achieved by placing the larvae in a 35 mm imaging dish (MolBiTec, Imaging dish 1.5) which contained 500 μL of E3 medium with 168 mg/L MS-222 (Tricaine Mehanesulfonate, Sigma) for 10 min. After removing the solution, fish were side-embedded in the imaging dish using 500 μL of 0.75% low-melting temperature agarose (Sigma, A4018). Wide-field imaging was done using the Zeiss Axio Observer fluorescence microscope (LD A-Plan 10× objective, NA = 0.25), and confocal imaging was done using a Zeiss LSM 800 confocal fluorescence microscope (LD LCI Plan-Apochromat 25× objective, NA = 0.8).

### Zebrafish Startle Reflex Analysis

For the behavioral analysis, wild-type 4–6 dpf zebrafish larvae were placed individually into custom-built sound delivery system described in a previous publication.^36^ For acoustic stimulation, we used a 90 dB stimulus and recorded the subsequent behavioral responses using a high-speed camera (xiQ USB3 vision) at a frame rate of 500 fps using custom scripts written in Python. The camera was placed 30 cm above the sound delivery system. The latency of the startle reflex was quantified manually by using an LED flash (780 nm wavelength) in parallel with the time of stimulus delivery. Escape responses were quantified manually, where we considered a short-latency startle response between 2 and 16 ms and a long-latency startle response above 16 ms.

### *Ex Vivo* Labeling of Surgically Resected Human Parathyroid Adenomas

The part of the study involving human tissues has been registered at clinicaltirals.gov and can be retrieved under the ID: NCT03831620. The study was approved by the ethics commission of canton Bern, Switzerland (KEKBE 2018–02218). Written informed consent was obtained from all patients. Human parathyroid tissues were surgically resected and were immediately put on dry ice and stored at −80 °C. The tissues were thawed at 4 °C and were washed with PBS thoroughly. The labeling of the tissues was achieved using 500 nM EvoSiR4 for 30 min in PBS containing 0.1% BSA. After labeling, the tissues were washed twice with PBS containing 0.1% BSA. For evocalcet competition experiments, the tissues were coincubated with EvoSiR4 and evocalcet in a 1:5 molar ratio for 30 min and then washed twice with PBS containing 0.1% BSA. The images were acquired using an IVIS Spectrum *in vivo* imaging system with a λ_ex_ of 780 nm and λ_em_ of 840 nm for the autofluorescence (ICG channel) and with a λ_ex_ of 650 nm and λ_em_ of 670 nm for the EvoSiR label (Cy5 channel).

### Statistics and Data Analysis

All statistical analysis was carried out in Python 3.9 using the SciPy package. Sample sizes (*n*) represent experiments conducted on independent days and each depicts an average value of three technical replicates, unless otherwise specified. Data shown are from at least 3 independent experiments and are represented as mean ± standard deviation, unless otherwise indicated. Statistical significance was determined using the Mann–Whitney test with the Bonferroni correction where applicable unless otherwise stated (*p < 0.05, **p < 0.01, ***p < 0.001, ****p < 0.0001). No statistical tests were applied to predetermine sample sizes, but our sample sizes were similar to those of previous reports.

## Results

### Selection of Optimal CaSR Ligands for the Incorporation of Fluorescent Moieties

**Figure 1:**
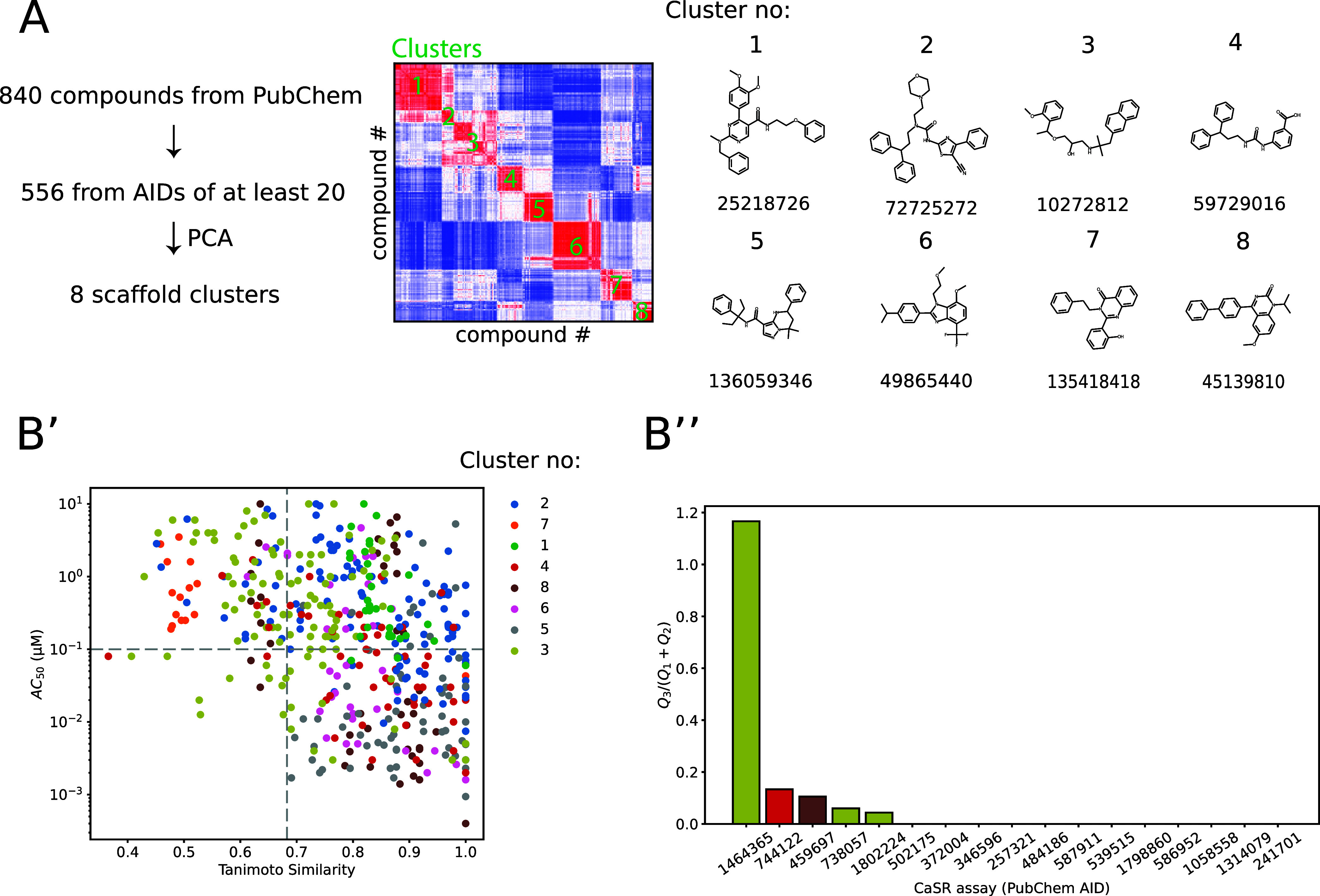
*In silico* analyses reveal optimal chemical scaffolds for the functionalization with fluorophores. (A) Description of the workflow to obtain 2D structure-based clusters on CaSR-binding molecules downloaded from PubChem. The heatmap depicts the correlation matrix of the Tanimoto-similarity of 10 latent dimensions of the 2D fingerprints for all compounds, which identified eight chemical clusters (green text). On the right, the seven most representative molecules and their PubChem ID from each chemical cluster are shown (closest Euclidean distance to the coordinates of cluster centroids). (B′) Scatter plot depicting the relationship between the Tanimoto similarity of each molecule to the most potent molecule of each assay (AID) and the half-maximal activity value (AC_50_). (B″) The ratio between the number of highly potent (<100 nM) and dissimilar molecules (*Q*_3_) to molecules with reduced potency (>100 nM) and high (*Q*_1_) or low (*Q*_2_) similarity is quantified. The assay with the AID 1464355 had the highest number of dissimilar and potent molecules.

To decipher the structural diversity of CaSR-binding molecular scaffolds, a total of 840 unique molecules were gathered *in silico* from PubChem activity assays (AID) with their empirical active concentration (AC_50_) values toward CaSR. Activity assays that contained at least 20 molecules were selected, which resulted in a total of 556 molecules. The dimensionality of the 881-bit PubChem substructure fingerprints of the molecules was reduced to 20 latent dimensions using principal component analysis (PCA). The first 10 PCs could explain 73% of the variance present in the data (Figure S1). We next calculated the correlation matrix from the 10 PCs and clustered the structures using the *k*-means clustering algorithm. The optimal number of clusters was eight, which was calculated from the inertia values (Figure S1). The chemical structures of the molecules closest to the cluster centers are listed in Figure 1. Next, we assessed which molecular scaffolds are most tolerant toward structural modifications without losing potency. To this end, within each AID, the AC_50_ value of each molecule was plotted as a function of the Tanimoto similarity value to that of the most potent molecule (Figure 1). The number of molecules within the quadrant with dissimilar structures and low AC_50_ values (*Q*_3_) normalized to the molecules in the quadrant with dissimilar structures and high AC_50_ values (*Q*_1_) and similar structures and high AC_50_ values (*Q*_2_) were quantified. Only 5 AIDs (459797, 738057, 744122, 1464365, and 1802224) showed a positive value, whereas AID 1464365 had the highest value, suggesting that this scaffold might be tolerant toward the incorporation of fluorescent moieties.

### Synthesis of EvoSiR

**Scheme 1:**
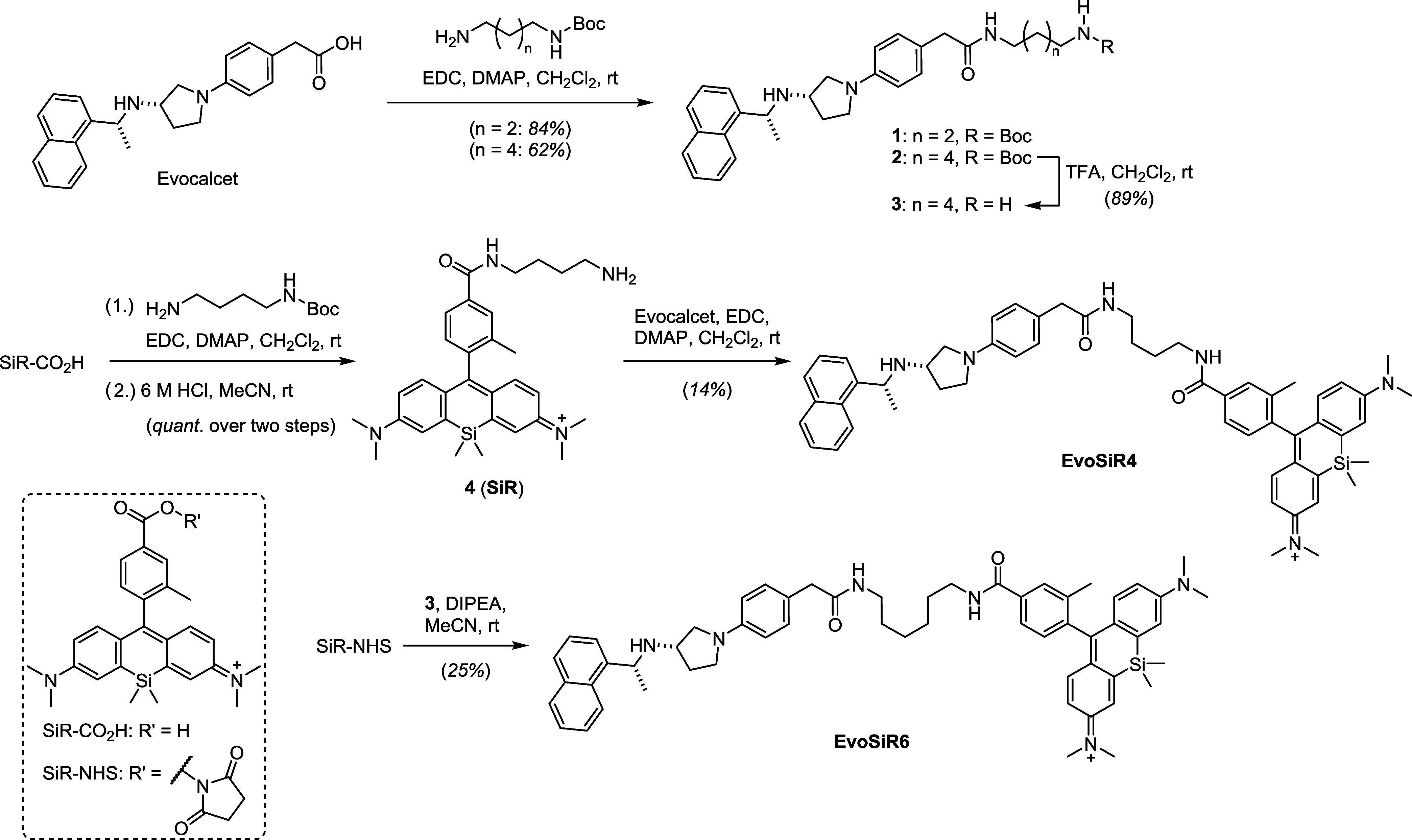
Synthesis of EvoSiR4 and EvoSiR6 Synthesis of the silicon-rhodamine conjugate CaSR probes EvoSiR4 and EvoSiR6. Abbreviations: Boc, *tert*-butyloxycarbonyl; EDC, 1-ethyl-3-(3-dimethylaminopropyl)carbodiimide; DMAP, 4-(dimethylamino)pyridine; TFA, trifluoroacetic acid; DIPEA, diisoproplyethylamine; NHS, *N*-hydroxysuccinimide.

Based on the *in silico* data and reported cryo-EM structures,^21,22^ evocalcet was extended at the carboxyl end with linkers prior to functionalization with SiR (Scheme 1). Using 1-ethyl-3-(3-(dimethylamino)propyl)carbodiimide (EDC) in the presence of DMAP efficiently coupled monoprotected bis-amines to deliver the corresponding amides **1** and **2**. Cleavage of the Boc-protecting group of **2** by treatment with TFA gave free amine **3**, which was reacted with the activated dye ester SiR-NHS to yield the final probe EvoSiR6. For EvoSiR4, we found it more advantageous to first couple the monoprotected bis-amine linker with silicon-rhodamine (SiR-CO_2_H). After Boc-deprotection, the free amine **4** was subsequently reacted with evocalcet with the aid of EDC and DMAP. The relatively low yields of the final probes EvoSiR4 and EvoSiR6 reflect their challenging chromatographic purification rather than the efficiency of the amide coupling steps. The isolation of the final probes may be optimized in future endeavors.

### EvoSiR Probes Have Improved CaSR Affinity Compared to Evocalcet in a Functional *In Vitro* Assay

**Figure 2:**
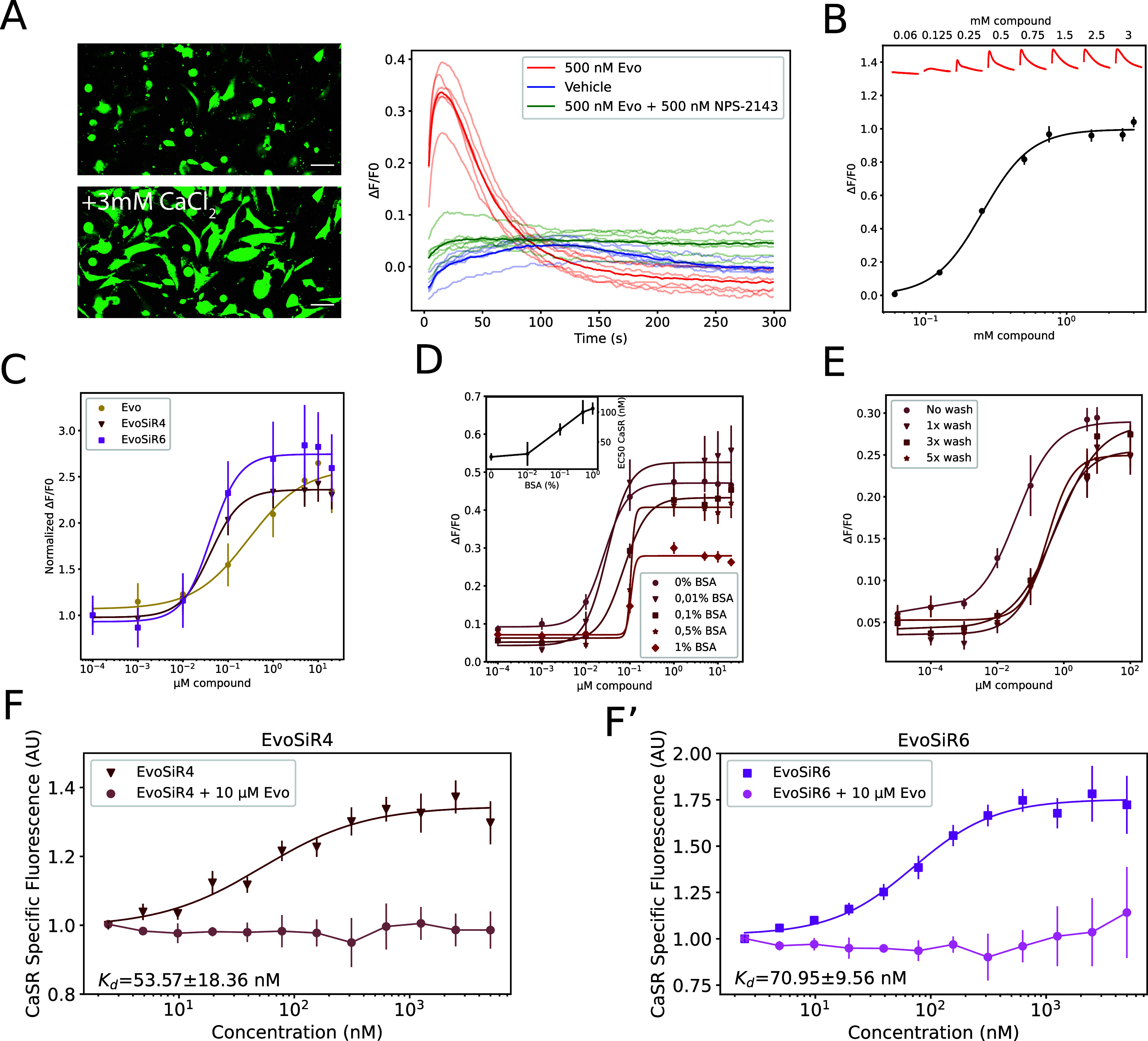
*In vitro* functional CaSR assay and binding experiments reveal improved potency of EvoSiR probes. (A) Left: Example images showing the increase of intracellular calcium in CCL39 cells overexpressing the human CaSR (HCAR) in response to the addition of 3 mM CaCl_2_ stained with Fluo-8 AM (white scale indicates 15 μm). Right: representative intracellular calcium transients measured by FLIPR in HCAR cells upon the addition of 0.5 mM calcium and either 500 nM evocalcet (Evo), vehicle, and 500 nM evocalcet + 500 nM CaSR inhibitor NPS-2143. (B) Concentration–response curve of intracellular calcium release in response to extracellular calcium in HCAR cells. CaSR activity was measured indirectly as a relative increase in fluorescence (d*F*/*F*_0_) (EC_50_ = 0.252 ± 0.01 mM, *n* = 3, average of triplicates). Representative traces for each concentration in mM are shown at the top of the plot in red. (C) Concentration–response curve of evocalcet and the two EvoSiR derivatives (EvoSiR4, EvoSiR6) on the positive allosteric modulation of CaSR activity (*n* = 5–13, average of triplicates and EC_50_ = 0.243 ± 0.015, 0.038 ± 0.009, and 0.042 ± 0.011 μM for evocalcet, EvoSiR4, and EvoSiR6, respectively). (D) Impact of various concentrations of bovine serum albumin (BSA) on the potency of EvoSiR4 on CaSR activity (*n* = 5, average of triplicates for all conditions). The inset indicates the EC_50_ values of each condition as a function of BSA percentage. (E) Impact of washing after a 5 min incubation of HCAR cells with various concentrations of EvoSiR4 and 0.1% BSA (*n* = 3, average of triplicates for all conditions, EC_50_ = 0.037 ± 0.008 (no wash), 0.38 ± 0.24 (1× wash), 0.62 ± 0.31 (3× wash), and 0.33 ± 0.21 (5× wash)). (F) Plot depicting the concentration dependence of CaSR binding estimated by the differential fluorescence intensity between the CCL39 (CaSR−) cells (nonspecific label) and HCAR (CaSR+) cells (specific label) with EvoSiR4 (F, *n* = 4, average of quadruplicates) and EvoSiR6 (F′, *n* = 3, average of quadruplicates). Preincubation of the cells with 10 μM evocalcet prior to incubation in EvoSiR compounds completely abolished the fluorescence difference between CaSR+ and CaSR– (*n* = 3 for both EvoSiR4 and EvoSiR6, in quadruplicates).

To assess CaSR receptor binding affinity, we quantified the concentration response of the PAM effect of the synthesized molecules and evocative effect on CaSR activity using an *in vitro* FLIPR cellular assay (Figure 2). In our assay, for evocalcet an EC_50_ value of 243 ± 15 nM in response to the addition of 0.5 mM CaCl_2_ was obtained. In comparison, both EvoSiR4 and EvoSiR6 exhibited a 6-fold improvement in potency compared to evocalcet with EC_50_ values in the range of 40 nM (Figure 2), whereas the EC_50_ value for evocalcet with only the linker (compound **1**, Scheme 1 and Figure S2) was 101 ± 6 nM (Figure S2). Importantly, the fluorescence properties of the EvoSiR probes at the concentrations used did not interfere with the assay fluorescent readout (data not shown). We next modeled the impact of albumin binding on the EC_50_ values for EvoSiR4 by adding different concentrations of bovine serum albumin (BSA). The addition of EvoSiR in 0.1% BSA increased the EC_50_ value to 64 ± 12 nM, 0.5% BSA to 102 ± 34 nM, and 1% BSA to 106 ± 42 nM (Figure 2), indicating saturable BSA binding. Similarly, the hill coefficients of the fitted curves also increased upon addition of BSA. In agreement, washing the molecules incubated in 1% BSA with HBSS upon 10 min incubation in the cellular assay led to a 10-fold reduction in the EC_50_ value (Figure 2). To estimate CaSR binding affinity, we quantified the differential fluorescence intensity between CaSR+ and CaSR– cells upon labeling with the EvoSiR probes. The dissociation constant (*K*_d_) was 53.57 ± 18.36 nM and 70.95 ± 9.56 nM for EvoSiR4 and EvoSiR6, respectively, which was comparable to the EC_50_ values measured in the functional assay (Figure 2). The CaSR-specific increase in relative fluorescence was confirmed by preincubating the cells in 10 μM evocalcet, which completely abolished this effect (Figure 2F). Next, we assessed the acute toxicity of the EvoSiR probes by measuring the viability of CaSR expressing (HCAR) and CaSR negative (CCL39) cells upon exposure to the compounds for 72 h. For evocalcet, no effect on viability was detected below a 50 μM concentration. In contrast, both EvoSiR4 and EvoSiR6 significantly reduced the viability of HCAR and CCL39 cells at 50 μM. The IC_50_ value of EvoSiR4 was 27.3 ± 2.3 and 36.0 ± 3.8 μM for HCAR and CCL39, respectively, and the IC_50_ value of EvoSiR6 was 26.0 ± 2.3 and 36.0 ± 6.0 μM for HCAR and CCL39, respectively. For both cells, no effect on the viability was apparent below 12.5 μM for EvoSiR probes. The results are depicted in Figure S3.

### Photophysical Properties of EvoSiR Probes

**Figure 3:**
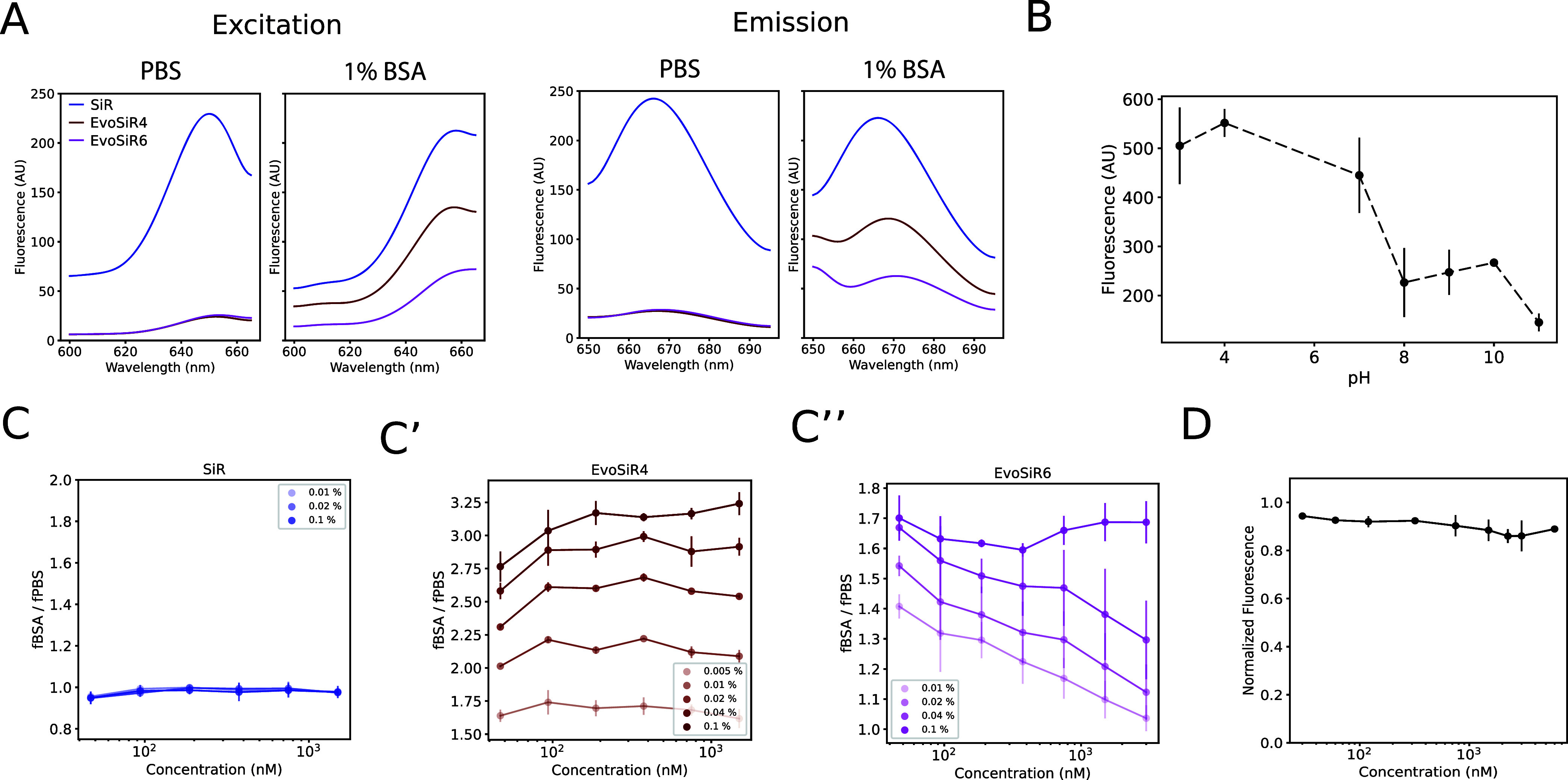
Photophysical properties of EvoSiR and the effect of BSA binding. (A) Plot depicting the fluorescence excitation and emission spectrum of SiR (**4**), EvoSiR4, and EvoSiR6. The peak excitation wavelength (λ_ex_) was 655 nm, and the peak emission wavelength (λ_em_) was 665 nm for all compounds. The addition of 1% BSA resulted in a 400% and 250% increase in the absolute fluorescence for EvoSiR4 and EvoSiR6, respectively, and had no effect on the absolute fluorescence of SiR (**4**). (B) Effect of pH on the peak fluorescence at 670 for 750 nM EvoSiR4 measured in PBS solution (*n* = 3 each). The absolute fluorescence was highest at below physiological pH, while pH above 10 resulted in a 50% decrease in fluorescence. (C) Effect of BSA binding on the absolute peak fluorescence of SiR (**4**) and EvoSiR probes using various concentrations of BSA. For the analysis, we used 30–1500 nM SiR (**4**) (C′), EvoSiR4 (C″), and EvoSiR6 (C″′) (*n* = 3 each, average of triplicates). The values on the *y* axis represent the absolute fluorescence value in BSA-containing PBS solution normalized to the absolute fluorescence value in PBS. (D) Plot depicting the effect of preincubating the BSA-containing PBS solution in various concentrations of evocalcet (30–6000 nM) on the fluorescence value of 350 nM EvoSiR4 (*n* = 6, average of triplicates). No significant decrease in the fluorescence was observed, indicating that EvoSiR4 and evocalcet occupy distinct binding sites on BSA.

For SiR (**4**), EvoSiR4, and EvoSiR6, standard photophysical parameters were determined, including extinction coefficients (ε), peak excitation (λ_ex_) and emission (λ_em_) wavelengths (Figure 3), quantum yields (Φ), and fluorescence lifetimes (τ), and are reported in Table 1. Regression curves for the analysis are shown in the Supporting Information in Figure S4.

**Table 1:** Photophysical Properties of EvoSiR Compounds

compound	ε (M^–1^ cm^–1^)	λ_ex_ (nM)	λ_em_ (nM)	Φ	τ (ns)
SiR (**4**)	7051	650	670	0.347	1.92 ± 0.733
EvoSiR4	14,175	650	670	0.288	2.43 ± 0.164
EvoSiR6	16,601	650	670	0.278	2.33 ± 0.135

### Fluorogenicity and BSA Binding of EvoSiR Probes

We next assessed the fluorogenicity of the probes by measuring the effects of pH and BSA binding using spectrofluorometry. In PBS, the absolute fluorescence of SiR (**4**) was 10 times higher than those of EvoSiR4 and EvoSiR6. The addition of 1% BSA significantly increased (400 and 250% for EvoSiR4 and EvoSiR6, respectively) the fluorescence of EvoSiR probes compared to PBS, reflecting fluorogenicity upon BSA binding, while it had no effect on SiR (**4**) (Figure 3). We found that the absolute fluorescence of the EvoSiR probes in PBS was strongly dependent on the pH of the solution with increasing fluorescence values in acidic conditions (Figure 3). In measuring the impact of protein binding on absolute fluorescence, we found no significant change in the absolute fluorescence of SiR (**4**) in response to the addition of BSA in the range of 0.01–0.1% (Figure 3). In contrast, in 0.1% BSA, EvoSiR4 and EvoSiR6 had 4-fold and 1.8-fold increased fluorescence compared to PBS, respectively. For EvoSiR4, the BSA-dependent increase in fluorescence was uniform in the concentration range tested (30–1500 nM), whereas for EvoSiR6, this was only observed at 0.1% BSA (Figure 3), suggesting a higher BSA binding ability of EvoSiR4. Next, we preincubated the BSA solution with various concentrations of evocalcet. We found no significant difference in the absolute fluorescence between the evocalcet preincubated samples and the control, suggesting that EvoSiR and evocalcet occupy different binding sites in BSA due to the bulky SiR moiety (Figure 3).

### Validation of Specificity of EvoSiR in a CaSR Overexpressing Cell Line

**Figure 4:**
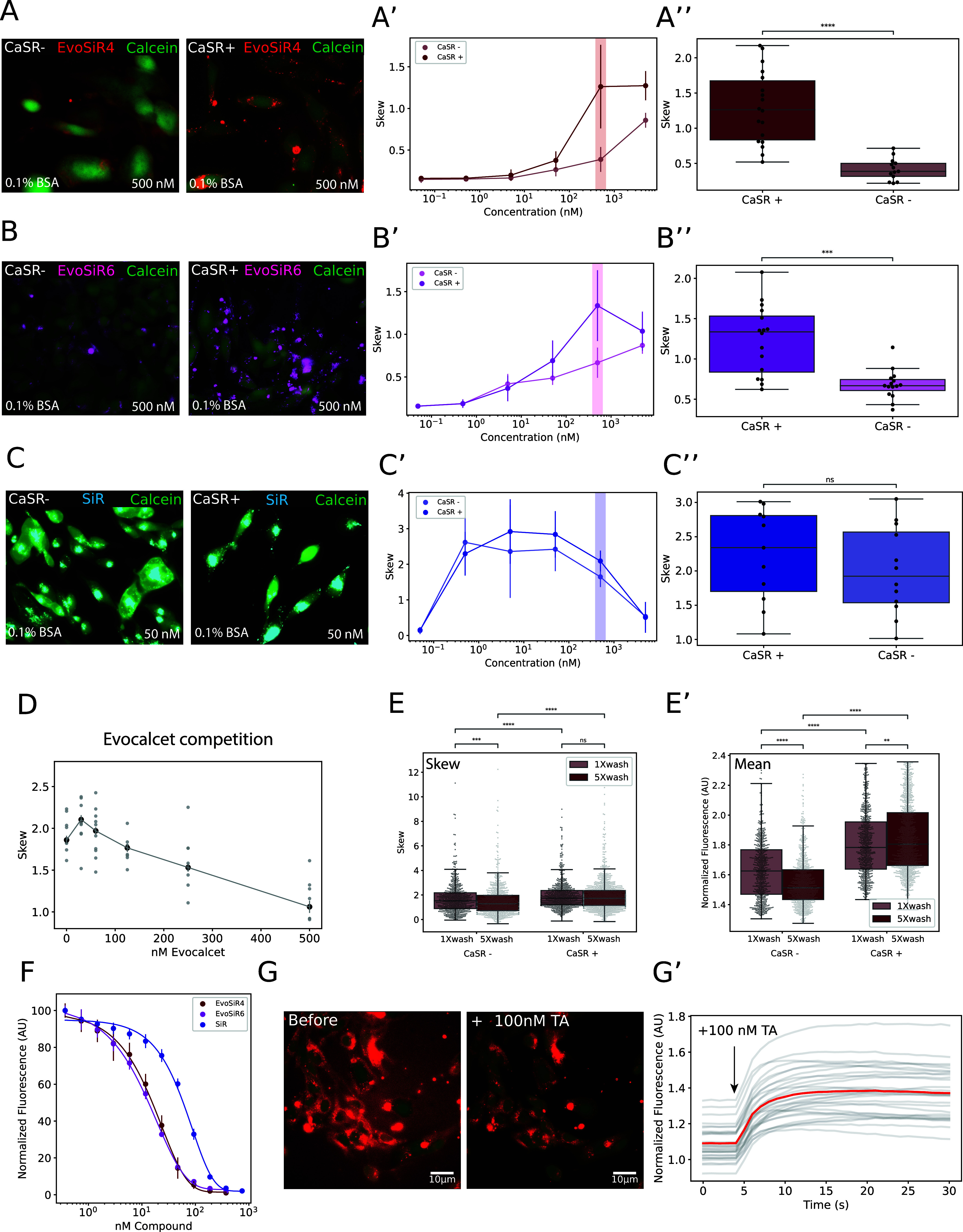
EvoSiR probes specifically label CaSR expressing cells. (A–C) Representative images of labeling with 500 nM of the probe of control (CaSR−) and CaSR expressing cells (CaSR+). Viable cells were identified by staining with Calcein AM. The middle plots (A′, B′, C′) depict the concentration–response effect measuring the skewness of the pixel intensity distribution for each cell (*n* = 9–16 independent experiments with three images per experiment for each condition). The right plots (A″, B″, C″) show the skew value for each compound at the 500 nM condition, each dot representing an independent experiment (average value of three images) (*p* < 0.0001, *p* = 0.0003 0.37, for EvoSiR4, EvoSiR6, and SiR (**4**), respectively; Mann–Whitney Wilcoxon). (D) Preincubating the CaSR+ cells with various concentrations of the nonfluorescent CaSR-positive allosteric modulator, evocalcet, which resulted in a concentration-dependent reduction of the EvoSiR4 labeling. Dots represent the mean value of three images for independent experiments (*n* = 6 for all conditions). (E) Boxplots depicting the differential effects of washout with 0.1% BSA on the CaSR– and CaSR+ cells. The left plot depicts the skewness of each cell (*n* = 3, 17–25 wells per condition, dots represent individual cells; *p* = 0.99 for the comparison of CaSR+ 1× wash and CaSR+ 5× wash, *p* = 0.00012 for the comparison of CaSR– 1× wash and CaSR– 5× wash, *p* < 0.0001 for all other comparisons, Mann–Whitney Wilcoxon test with Bonferroni correction), whereas the right plot depicts the average normalized pixel intensity of the same experiments (*p* = 0.008 for the comparison of CaSR+ 1× wash and CaSR+ 5× wash, *p* < 0.0001 for all other comparisons, Mann–Whitney Wilcoxon test with Bonferroni correction). (F) Concentration–response curve of the fluorescence quenching for 750 nM SiR probes by the administration of tannic acid measured with a spectrofluorometer. The EC_50_ values are 15.59 ± 4.32, 14.95 ± 2.65, and 58.17 ± 4.86 nM for EvoSiR4, EvoSiR6, and SiR (**4**), respectively (*n* = 3 independent experiments, average of triplicates for each condition). (G) On the left, the two representative images show the effect of administration of 100 nM tannic acid on the signal-to-noise ratio of EvoSiR4 labeling on CaSR+ cells. On the right, the plot depicts the 40% increase in signal-to-noise upon the addition of 100 nM tannic acid in the representative time lapse imaging experiment, where the red line indicates the mean signal-to-noise ratio and the gray line indicates each cell (*n* = 31 cells from a single experiment).

*In vitro* labeling of human CaSR stably overexpressing HCAR cells (CaSR+) with EvoSiR4 and EvoSiR6 resulted in highly fluorescent membrane-associated puncta, which were largely absent in the labeling of the nontransfected CCL39 control cells (CaSR−) (Figure 4). We therefore used the skewness of the pixel intensity distribution for the evaluation of labeling specificity. For EvoSiR4, a significant increase in skewness was measured for HCAR cells at concentrations above 500 nM with no BSA and 0.1% BSA, and for concentrations above 2500 nM with 1% BSA compared to those of CCL39. With 0.1% BSA, the saturation of the skewness values appeared at 500 nM for HCAR (CaSR+) but was not saturated for the CCL39 (CaSR−) cells even at 5000 nM with a 3.7-fold increase in skewness for HCAR cells at 500 nM (Figure 4). We repeated the same experiment with the EvoSiR6 probe, which similarly differentiated between the CaSR+ and CaSR– cells, resulting in a 2.3-fold increase in skewness for CaSR+ cells at 500 nM, confirming the specific labeling (Figure 4). Incubation with SiR (**4**) revealed no significant difference between CaSR+ and CaSR– cells, confirming the specificity of EvoSiR4 CaSR labeling (Figure 4). Assuming identical binding sites of evocalcet and EvoSiR probes and to confirm the labeling specificity, we conducted a competition experiment by preincubating the CaSR+ cells with 0–500 nM evocalcet in 0.1% BSA. Evocalcet concentration-dependently decreases the highly fluorescent puncta, resulting in a significant, 55% reduction in the skewness of the pixel intensity distribution in CaSR overexpressing cells (Figure 4). Washing with BSA showed a divergent effect on the CaSR+ and CaSR– cells, leading to an increased signal background ratio. In the CaSR– cells, washing 5 times with 0.1% BSA resulted in a 21 and 10% reduction of the skewness and the normalized mean pixel intensity, respectively, reflecting the washout of non-CaSR labeling (*i.e.*, background). In contrast, washing the CaSR+ cells with BSA had no effect on the skewness (median of 1.739 and 1.732 for 1× and 5× wash, respectively) and resulted in a marginal but significant increase (108%) of the normalized mean pixel intensity (Figure 4). To evaluate the long-term effects of CaSR labeling, we incubated CaSR– and CaSR+ cells at concentrations ranging from 50 nM to 6250 nM EvoSiR4 probe for 72 h (Figure S5). Using wide-field microscopy, we observed highly fluorescent puncta in the CaSR+ cells similar to the acute incubation, which were almost completely absent in the CaSR– cells, resulting in a statistically significant, 1.3-fold and 2.5-fold mean fluorescence difference between the CaSR+ and CaSR– cells for the 50 nM and the 200 nM condition, respectively (Figure 4). Next, we serendipitously discovered that tannic acid is a highly efficient molecular quencher of the fluorescence of EvoSiR probes and SiR (**4**) with IC_50_ values in the low nanomolar ranges (Figure 4). We therefore tested whether tannic acid can be used as a contrast-enhancing agent for EvoSiR labeling. As shown in Figure 4, in CaSR+ cells labeled with 500 nM EvoSiR4, the administration of 100 nM tannic acid indeed resulted in a 40% increase in the signal-to-noise ratio (Figure 4), suggesting that tannic acid quenches the CaSR-bound EvoSiR4 less efficiently.

### EvoSiR4 and EvoSiR6 Specifically Label Hair Cells of Neuromasts of the Zebrafish Lateral Line Organ *In Vivo*

**Figure 5:**
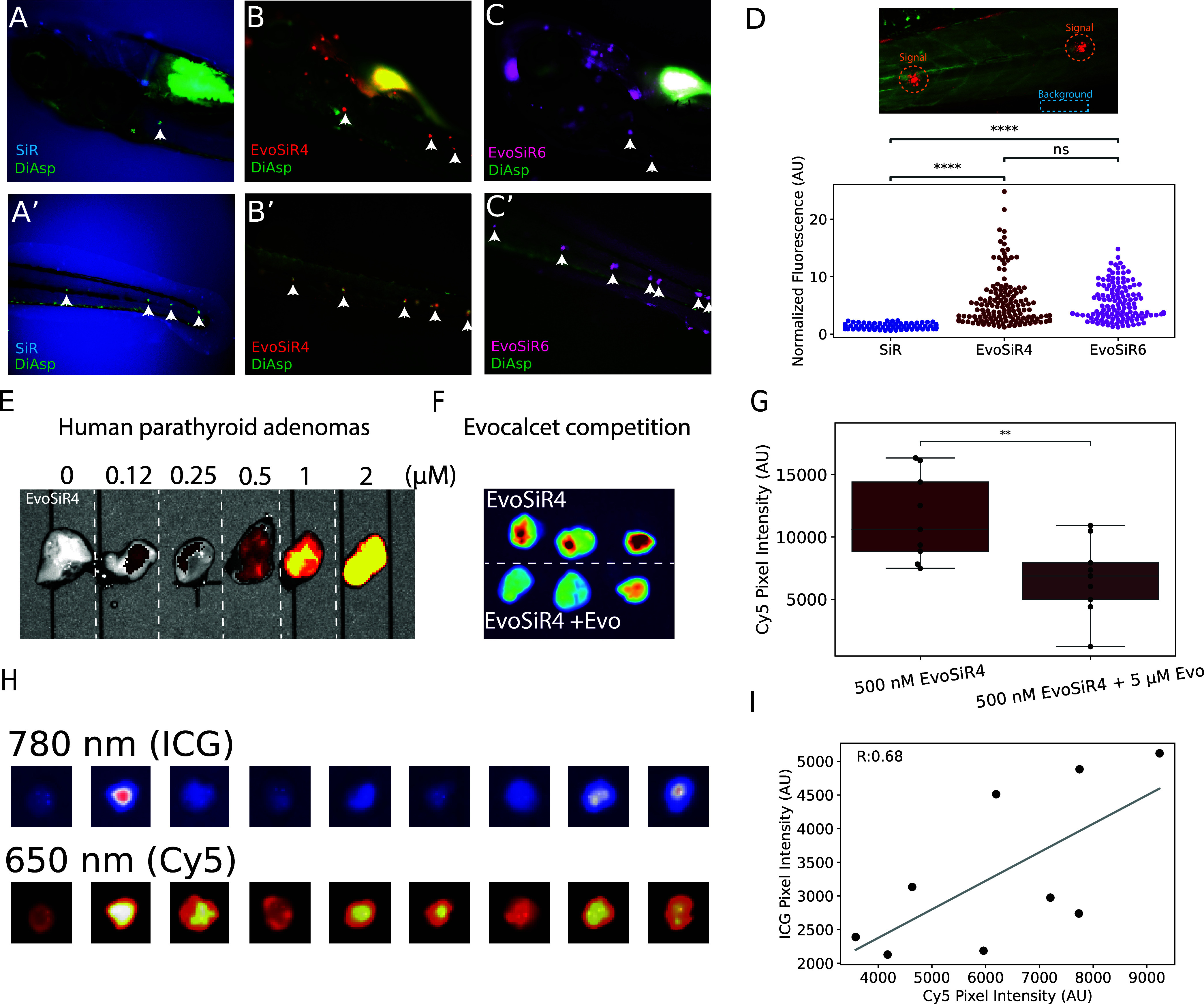
EvoSiR probes specifically label neuromasts in zebrafish larvae. (A–C) Representative images of live zebrafish larvae costained with SiR (**4**) or EvoSiR probes and the specific neuromast label, DiAsp. (D) The top image shows the EvoSiR4 labeling of neuromasts acquired by confocal microscopy. The bottom plot shows the normalized fluorescence of the neuromasts identified with the DiAsp staining to the background fluorescence value for each probe. Each dot represents the average normalized pixel intensity value of a neuromast with a mean value and standard deviation of 1.28 ± 0.41, 5.71 ± 4.4, and 5.6 ± 3.1 for SiR (**4**), EvoSiR4, and EvoSiR6, respectively (*n* = 18, 17, and 14 individual fish for SiR(**4**), EvoSiR4, and EvoSiR6, respectively, *n* = 125, 147, and 130 neuromasts for SiR(4), EvoSiR4, and EvoSiR6, respectively; *p* = 0.64 for comparing EvoSiR4 and EvoSiR6, *p* < 0.0001 for all other comparisons, Mann–Whitney Wilcoxon test with Bonferroni correction). (E) Concentration dependence of EvoSiR4 labeling on *ex vivo* human parathyroid adenomas. At 650 nm excitation, no autofluorescence of the tissue is observed, whereas the addition of >500 nM EvoSiR4 results in a marked increase in tissue fluorescence. (F) Representative images of three parathyroid adenomas cut in half, where the top halves were labeled with 500 nM EvoSiR4, whereas the bottom halves co-administered with 500 nM EvoSiR4 and 2.5 μM evocalcet. A significant, 64% decrease in tissue fluorescence was observed upon evocalcet competition. (G) Boxplot depicting the results of evocalcet competition experiments on nine parathyroid adenomas (*p* = 0.006, *n* = 9 individual adenomas halved for the two conditions, Student’s *t* test). (H) Representative images of the autofluorescence at 780 nm (ICG channel) and EvoSiR at 650 nm (Cy5 channel) of nine labeled, surgically resected *ex vivo* human parathyroid adenomas. (I) Plot depicting a significant positive correlation between the average pixel intensity of the Cy5 channel (EvoSiR) and the ICG channel (autofluorescence) (*n* = 9 individual adenomas, Pearson’s *R* = 0.676, *p* = 0.04).

To confirm the specificity of EvoSiR probes in an *in vivo* setting, we next assessed labeling specificity of hair cells of the neuromasts within the lateral line organ of 4–6 days post fertilization (dpf) larval zebrafish, which has been previously confirmed to highly express CaSR.^37^ As expected, upon addition of either EvoSiR4 or EvoSiR6, we observed a marked, 5.7-fold and 5.6-fold increase in the fluorescence intensity within the neuromasts, respectively, whose identify we confirmed pharmacologically by co-labeling with the hair cell-specific dye DiAsp^35^ (Figure 5). This increase in fluorescence for the EvoSiR probes was on average 4.4-fold higher compared to SiR (**4**), which also labeled other cells and exhibited an approximately 4-fold higher background fluorescence (5298 ± 2367 AU, 1339 ± 803 AU, and 1715 ± 580 AU for SiR (**4**), EvoSiR4, and EvoSiR6, respectively). In agreement with their comparable CaSR EC_50_ and *K*_d_ values, no significant difference between EvoSiR4 and EvoSiR6 was observed (Figure 5).

### EvoSiR Had No Effect on the Modulation of Startle Reflex in Zebrafish Larvae

To investigate whether EvoSiR can penetrate into the brain of zebrafish larvae, we quantified the latency of the startle reflex in response to acoustic-vibrational stimuli, which has been previously reported to be modulated by CaSR.^10,38^ In response to abrupt low-level acoustic stimulation, zebrafish elicited the startle reflex which comprised short-latency responses (2–12 ms) in 12%, long-latency responses (>12 ms) in 3%, and no response in 85% of the total number of stimulations. Bath-applied EvoSiR4 appeared to have no effect on the basic locomotion capabilities of the larvae at up to 10 μM; however, it was lethal beyond 20 μM. We found no significant difference in either the percentage of elicited startle responses or the ratio of short-latency escapes (Figure S6). To assess target engagement of EvoSiR4 in the brain, we imaged the transgenic *Tol056* zebrafish strain, which express eGFP in the Mauthner-neurons in the hindbrain responsible for the initiation of the short-latency escape response using confocal microscopy.^36^ In agreement with the behavioral data, we found no significant EvoSiR label, indicating that the bath-applied probe does not enter the zebrafish CNS (Figure S6).

### EvoSiR Labeling of *Ex Vivo* Human Parathyroid Adenomas

To assess the utility of EvoSiR in labeling CaSR expressing human tissues, we applied EvoSiR4 to *ex vivo* surgically resected human parathyroid adenomas, which showed a dose-dependent increase in tissue fluorescence at 650 nm upon the addition of EvoSiR4 (Figure 5). We next conducted competition experiments in which tissues were halved and one-half was coincubated with EvoSiR4 and evocalcet. We observed a significant (64%) decrease in the absolute fluorescence in the evocalcet-competed tissues (Figure 5). Next, we stained nine additional adenomas and compared the labeling intensity with the autofluorescence of the tissues at 780 nm. The intensity of near-infrared (NIR) autofluorescence and CaSR expression of the parathyroid glands have been shown to be significantly lower in adenomas compared to the normal parathyroid glands.^39^ We acquired images at 780 and 650 nm in parallel, which showed a significant positive correlation (Pearson’s *R* = 0.68) between the autofluorescence and the EvoSiR4 labeling, which might indicate that EvoSiR4 remains specific toward CaSR in human tissues (Figure 5).

## Discussion

Mounting transdisciplinary research interest in the interrogation of CaSR signaling warrants the development of fast, broadly applicable, and versatile molecular tools for its visualization and the perturbation of its function. In the current study, we describe the design, synthesis, and biological characterization of novel SiR functionalized CaSR targeting fluorescent probes of evocalcet called EvoSiR. The *in silico* and *in vitro* data show that evocalcet is tolerant toward the introduction of large chemical tags at the carboxyl end, which renders the potently CaSR interacting chemical scaffold optimal for the development of chemical probes. Using spectrophotometric approaches, we demonstrate that EvoSiR probes are not only potent CaSR ligands but also efficiently bind BSA, enabling the removal of nonspecific signals. This was supported by efficient *in vitro* labeling and competition experiments on a CaSR overexpressing cell line. Furthermore, we confirmed the *in vivo* applicability of EvoSiR4 and EvoSiR6, which specifically labeled the hair cells in live zebrafish larvae, Lastly, we explored the utility of EvoSiR in labeling *ex vivo* human parathyroid adenomas without BSA washing, showing a strong correlation with parathyroid glands, and a 2-fold increase to the autofluorescence signal.

From the diverse small molecules that have been developed to target CaSR,^18^ the selection of a chemical scaffold suitable for the incorporation of fluorescent tags could be ambiguous. Indeed, our initial strategy of linking an NBD fluorophore to the meta-position of the *N*-benzyl group of quinazoline-2-ones, similar to the potent calcilytic compounds ATF936 and AXT914,^17^ resulted in diminished potency toward CaSR, in agreement with previously published SAR data,^40^ rendering the molecule inadequate for CaSR-specific labeling purposes (data not shown). In addition, these *N*-benzyl quinazoline-2-ones are very lipophilic compounds per se that ultimately resulted in considerable nonspecific labeling of the fluorescent NBD-conjugates. In contrast, SAR studies on the evocalcet structure provided evidence that the carboxyl position of the molecule is tolerant toward structural modifications. In agreement, recent Cryo-EM studies have shown that in the CaSR-bound state, the carboxyl position of evocalcet is less constrained within the neighboring environment.^21,22^ To acquire a comprehensive picture of potentially suitable chemical scaffolds, we evaluated all of the quantified structures from SAR studies available from PubChem. In accordance with the literature data on evocalcet, the analysis revealed this structure to be the least sensitive to structural modifications, which led us to investigate the impact of fluorophore conjugation on its potency and specificity on CaSR.

Using a functional CaSR assay and *in vitro* labeling experiments, we showed that EvoSiR improves with a 6-fold increase on CaSR activity compared to evocalcet (EC_50_ = 243 nM), with an EC_50_ value in the low nanomolar ranges (<50 nM) and remains specific toward the receptor below 500 nM. Binding experiments showed comparable *K*_d_ values (<70 nM) to the EC_50_ values in the functional assay, whereas competition experiments on CaSR binding sites with evocalcet confirmed specific labeling. As shown in a previous report in which the SAR of evocalcet structures was evaluated, several analogues modified at the carboxyl position exhibited higher CaSR activity, whereas evocalcet had the most favorable PK profile with no direct CYP2D6 inhibition.^41^ Of note, the four-carbon linker conjugation to evocalcet (compound **1**, Scheme 1) also reduced the EC_50_ value to 101 nM. Therefore, it is plausible that the further optimization of the carboxyl position of evocalcet could yield molecules with even higher activity in the future.

Using spectrofluorometry, we revealed strong BSA binding of EvoSiR, which was consistent with the observed concentration-dependent decrease in CaSR affinity upon the addition of BSA in the functional assay. Albumin binding is a critical parameter to optimize the design of *in vivo* experiments and potential clinical translation and was different for the two EvoSiR probes EvoSiR4 and EvoSiR6. Two experimental results support the potential positive effect of BSA on the EvoSiR target engagement. First, the CaSR-specific labeling of cells resulted in an increased signal-to-background ratio. Second, our *in vivo* data on labeling the neuromasts of zebrafish larvae showed a superior signal-to-noise ratio of EvoSiR to existing labels (DiAsp), which suggests that the high albumin binding of the molecule may improve its labeling capacity. Still, it remains to be elucidated in a rodent model and in humans whether such high albumin affinity would result in diminished target engagement; or would improve the signal-to-noise ratio by washing out nonspecific weak binding interactions from the target site.

In the removal of solid tumors and the preservation of healthy tissues during surgery, the application of fluorescent dyes such as Indocyanine Green (ICG)^42^ and the protoporphyrin IX precursor 5-aminolevulinic acid (5-ALA)^43^ has notable clinical utility. However, the feasibility of applying always-on fluorescent probes in the fluorescence-guided surgery context, especially in the form of topical sprays can be impaired by low target specificity and high background fluorescence.^44^ Activatable fluorescent probes such as enzyme-cleavable probes or pH-activable probes showed promising results in the accurate determination of tumor margins *in situ*.^45,46^ Moreover, recent developments of multivariate AND-gate optical contrast agents, which produce a signal upon least at two proteolytic processes may further enhance the contrast between the tumor boundaries and surrounding healthy tissues.^47^

On the translational perspective, the visualization of CaSR in the human parathyroid glands could enhance the localization and thus preservation of the tissue, which is an integral objective during various head and neck surgical procedures. For example, during thyroidectomy, which is performed approximately 100,000 times annually in just the US, transient and persistent damage to the parathyroid glands represent a major complication in up to 30 and 3% of the cases, respectively.^48^ Currently the relatively high far-red autofluorescence of the parathyroid glands compared to the surrounding tissues is used for parathyroid gland identification, which does not inform surgeons about the perfusion and the vitality of the glands during the operation.^49^ For parathyroid gland perfusion, indocyanine green angiography can be applied, which is an aspecific label.^50^ Although the recently developed structure-inherent probe, T800-F that is specifically taken up by the parathyroid glands is chemically more compact, its flexibility to optimize for clinical use (DMPK, toxicity) might be limited.^51^ The application of small-molecule CaSR-specific probes like EvoSiR4 for fluorescence-guided thyroidectomy could therefore help in both PG identification and assessment of PG perfusion of the surrounding vasculature. EvoSiR probes exhibit nanomolar potency toward CaSR, ensuring specificity. Moreover, the probes are nontoxic below 10 μM and possess a high affinity for albumin, a characteristic that may facilitate the removal of EvoSiR not bound to CaSR during the washing process. Intriguingly, EvoSiR4 stably and specifically labeled CaSR even after 72 h incubation in live cells, probably because it is an allosteric modulator that does not influence the trafficking of this GPCR. Thus, allosteric probes could be better suited than orthosteric ligands for an *in vivo* CaSR labeling application. In addition, the fluorogenicity of the probe upon albumin binding may enable visualization of the vasculature of the PG, allowing the assessment of both the anatomy and the perfusion parameter with a single probe.

Our results also support the possibility of applying EvoSiR probes as an intraoperative topical spray for parathyroid gland identification *in vivo* in the future, because the highly potent SiR quencher tannic acid, identified in this study, may increase target-related fluorescence by selectively quenching only the EvoSiR not bound to CaSR. Our *in vitro* data support this possibility, as the addition of tannic acid significantly improved the signal-to-background ratio in cellular CaSR labeling experiments. Although the *in vivo* applicability of this technique may be challenging, EvoSiR probes have translational potential for the development of optimized probes that can inform surgeons on the anatomical identification of CaSR expressing parathyroid glands in excised tissues.^52^ Therefore, considering ADMETOX properties, the labeling capacity of EvoSiR probes will be explored in preclinical *in vivo* models.

## Conclusions

To our knowledge, the SiR conjugates of the carboxyl end of evocalcet (EvoSiR) are the first fluorescent CaSR probes that show low nanomolar CaSR activity and improved potency over evocalcet. The CaSR labeling capacity of EvoSiR probes was validated in various *in vitro*, *in vivo* settings and *ex vivo* human parathyroid tissues. The tolerance of evocalcet toward functionalization with fluorophores or other tags permits large-scale optimization for biomedical applications.

## Supporting Information Available

Properties of the PCA and k-means algorithms (Figure S1); dose-response curve of EvoL4 (**1**) (Figure S2); effects of EvoSiR probes and evocalcet on cell viability (Figure S3); quantum yield and fluorescence lifetime measurements (Figure S4); long-term (72 h) labeling of live CaSR expressing and CaSR negative cells with EvoSiR4 (Figure S5); effect of EvoSiR4 on zebrafish startle behavior (Figure S6); and compound synthesis including ^1^H, ^13^C NMR, and HPLC data (PDF)

## References

[ref1] Hofer A. M.; Brown E. M. Extracellular calcium sensing and signalling. Nat. Rev. Mol. Cell Biol. 2003, 4, 530–538. 10.1038/nrm1154. 12838336

[ref2] Bouschet T.; Henley J. M. Calcium as an extracellular signalling molecule: perspectives on the Calcium Sensing Receptor in the brain. C. R. Biol. 2005, 328, 691–700. 10.1016/j.crvi.2004.10.006. 16125647 PMC3310908

[ref3] Chen R. A.; Goodman W. G. Role of the calcium-sensing receptor in parathyroid gland physiology. Am. J. Physiol. Renal Physiol. 2004, 286, F1005–F1011. 10.1152/ajprenal.00013.2004. 15130894

[ref4] Vezzoli G.; Soldati L.; Gambaro G. Roles of calcium-sensing receptor (CaSR) in renal mineral ion transport. Curr. Pharm. Biotechno. 2009, 10, 302–310. 10.2174/138920109787847475. 19355940

[ref5] Hendy G. N.; Canaff L. Calcium-Sensing Receptor Gene: Regulation of Expression. Front. Physiol. 2016, 7, 394 10.3389/fphys.2016.00394. 27679579 PMC5020072

[ref6] Yarova P. L.; Stewart A. L.; Sathish V.; Britt R. D.; Thompson M. A.; Lowe A. P. P.; Freeman M.; Aravamudan B.; Kita H.; Brennan S. C.; Schepelmann M.; Davies T.; Yung S.; Cholisoh Z.; Kidd E. J.; Ford W. R.; Broadley K. J.; Rietdorf K.; Chang W.; Bin Khayat M. E.; Ward D. T.; Corrigan C. J.; Ward J. P. T.; Kemp P. J.; Pabelick C. M.; Prakash Y. S.; Riccardi D. Calcium-sensing receptor antagonists abrogate airway hyperresponsiveness and inflammation in allergic asthma. Sci. Transl. Med. 2015, 7, 284ra60 10.1126/scitranslmed.aaa0282. PMC4725057 25904744

[ref7] Iamartino L.; Brandi M. L. The calcium-sensing receptor in inflammation: Recent updates. Front. Physiol. 2022, 13, 1059369 10.3389/fphys.2022.1059369. 36467702 PMC9716066

[ref8] Omori H.; Kawabata Y.; Yoshida Y.; Nagamoto Y.; Kawabata F.; Nishimura S.; Tabata S. Oral expressions and functional analyses of the extracellular calcium-sensing receptor (CaSR) in chicken. Sci. Rep 2022, 12, 17762 10.1038/s41598-022-22512-6. 36273034 PMC9588031

[ref9] Ruat M.; Traiffort E. Roles of the calcium sensing receptor in the central nervous system. Best Pract. Res., Clin. Endocrinol. Metab. 2013, 27, 429–442. 10.1016/j.beem.2013.03.001. 23856270

[ref10] Shoenhard H.; Jain R. A.; Granato M. The calcium-sensing receptor (CaSR) regulates zebrafish sensorimotor decision making via a genetically defined cluster of hindbrain neurons. Cell Rep. 2022, 41, 111790 10.1016/j.celrep.2022.111790. 36476852 PMC9813870

[ref11] Riccardi D.; Ward J. P. T.; Yarova P. L.; Janssen L. J.; Lee T. H.; Ying S.; Corrigan C. J. Topical therapy with negative allosteric modulators of the calcium-sensing receptor (calcilytics) for the management of asthma: the beginning of a new era?. Eur. Respir. J. 2022, 60, 2102103 10.1183/13993003.02103-2021. 35058244

[ref12] Iamartino L.; Elajnaf T.; Kallay E.; Schepelmann M. Calcium-sensing receptor in colorectal inflammation and cancer: Current insights and future perspectives. World J. Gastroenterol. 2018, 24, 4119–4131. 10.3748/wjg.v24.i36.4119. 30271078 PMC6158479

[ref13] Feng C.; Bao X.; Shan L.; Ling Y.; Ding Y.; Wang J.; Cao Y.; Wang Q.; Cui W.; Xu S. Calcium-Sensing Receptor Mediates β-Amyloid-Induced Synaptic Formation Impairment and Cognitive Deficits via Regulation of Cytosolic Phospholipase A2/Prostaglandin E2Metabolic Pathway. Front. Aging Neurosci. 2020, 12, 144 10.3389/fnagi.2020.00144. 32670047 PMC7328130

[ref14] Chu H.; Qin Z.; Ma J.; Xie Y.; Shi H.; Gu J.; Shi B. Calcium-Sensing Receptor (CaSR)-Mediated Intracellular Communication in Cardiovascular Diseases. Cells 2022, 11, 3075 10.3390/cells11193075. 36231037 PMC9562006

[ref15] Leach K.; Gregory K. J.; Kufareva I.; Khajehali E.; Cook A. E.; Abagyan R.; Conigrave A. D.; Sexton P. M.; Christopoulos A. Towards a structural understanding of allosteric drugs at the human calcium-sensing receptor. Cell Res. 2016, 26, 574–592. 10.1038/cr.2016.36. 27002221 PMC4856764

[ref16] Lang W.; Yuan C.; Zhu L.; Du S.; Qian L.; Ge J.; Yao S. Q. Recent advances in construction of small molecule-based fluorophore-drug conjugates. J. Pharm. Anal. 2020, 10, 434–443. 10.1016/j.jpha.2020.08.006. 33133727 PMC7591808

[ref17] Widler L. Calcilytics: antagonists of the calcium-sensing receptor for the treatment of osteoporosis. Future Med. Chem. 2011, 3, 535–547. 10.4155/fmc.11.17. 21526895

[ref18] Diao J.; DeBono A.; Josephs T. M.; Bourke J. E.; Capuano B.; Gregory K. J.; Leach K. Therapeutic Opportunities of Targeting Allosteric Binding Sites on the Calcium-Sensing Receptor. ACS Pharmacol. Transl. Sci. 2021, 4, 666–679. 10.1021/acsptsci.1c00046. 33860192 PMC8033781

[ref19] Mueller J. P. J.; Dobosz M.; O’Brien N.; Abdoush N.; Giusti A. M.; Lechmann M.; Osl F.; Wolf A.-K.; Arellano-Viera E.; Shaikh H.; Sauer M.; Rosenwald A.; Herting F.; Umaña P.; Colombetti S.; Pöschinger T.; Beilhack A. ROCKETS – a novel one-for-all toolbox for light sheet microscopy in drug discovery. Front. Immunol. 2023, 14, 1034032 10.3389/fimmu.2023.1034032. 36845124 PMC9945347

[ref20] Akizawa T.; Ikejiri K.; Kondo Y.; Endo Y.; Fukagawa M. Evocalcet: A New Oral Calcimimetic for Dialysis Patients With Secondary Hyperparathyroidism. Ther. Apheresis Dial. 2020, 24, 248–257. 10.1111/1744-9987.13434. PMC7317959 31486206

[ref21] Gao Y.; Robertson M. J.; Rahman S. N.; Seven A. B.; Zhang C.; Meyerowitz J. G.; Panova O.; Hannan F. M.; Thakker R. V.; Bräuner-Osborne H.; Mathiesen J. M.; Skiniotis G. Asymmetric activation of the calcium-sensing receptor homodimer. Nature 2021, 595, 455–459. 10.1038/s41586-021-03691-0. 34194040 PMC8826748

[ref22] Chen X.; Wang L.; Cui Q.; Ding Z.; Han L.; Kou Y.; Zhang W.; Wang H.; Jia X.; Dai M.; Shi Z.; Li Y.; Li X.; Geng Y. Structural insights into the activation of human calcium-sensing receptor. eLife 2021, 10, e68578 10.7554/eLife.68578. 34467854 PMC8476121

[ref23] Grossenbacher P.; Essers M. C.; Moser J.; Singer S. A.; Häusler S.; Stieger B.; Rougier J.-S.; Lochner M. Bioorthogonal site-selective conjugation of fluorescent dyes to antibodies: method and potential applications. RSC Adv. 2022, 12, 28306–28317. 10.1039/D2RA05580E. 36320493 PMC9533196

[ref24] Helal K. Y.; Maciejewski M.; Gregori-Puigjané E.; Glick M.; Wassermann A. M. Public Domain HTS Fingerprints: Design and Evaluation of Compound Bioactivity Profiles from PubChem’s Bioassay Repository. J. Chem. Inf. Model. 2016, 56, 390–398. 10.1021/acs.jcim.5b00498. 26898267

[ref25] Lloyd S. Least squares quantization in PCM. IEEE Trans. Inf. Theory 1982, 28, 129–137. 10.1109/TIT.1982.1056489.

[ref26] Satopaa V.; Albrecht J.; Irwin D.; Raghavan B. In Finding a ″Kneedle″ in a Haystack: Detecting Knee Points in System Behavior, 2011 31st International Conference on Distributed Computing Systems Workshops; IEEE, 2011; pp 166–171.

[ref27] Bajusz D.; Rácz A.; Héberger K. Why is Tanimoto index an appropriate choice for fingerprint-based similarity calculations?. J. Cheminform. 2015, 7, 20 10.1186/s13321-015-0069-3. 26052348 PMC4456712

[ref28] Snare M. J.; Treloar F. E.; Ghiggino K. P.; Thistlethwaite P. J. The photophysics of rhodamine B. J. Photochem. 1982, 18, 335–346. 10.1016/0047-2670(82)87023-8.

[ref29] Uggeri J.; Gatti R.; Belletti S.; Scandroglio R.; Corradini R.; Rotoli B. M.; Orlandini G. Calcein-AM is a detector of intracellular oxidative activity. Histochem. Cell Biol. 2000, 122, 499–505. 10.1007/s00418-004-0712-y. 15503120

[ref30] Stringer C.; Wang T.; Michaelos M.; Pachitariu M. Cellpose: a generalist algorithm for cellular segmentation. Nat. Methods 2021, 18, 100–106. 10.1038/s41592-020-01018-x. 33318659

[ref31] Tanimoto M.; Ota Y.; Horikawa K.; Oda Y. Auditory input to CNS is acquired coincidentally with development of inner ear after formation of functional afferent pathway in zebrafish. J. Neurosci. 2009, 29, 2762–2767. 10.1523/JNEUROSCI.5530-08.2009. 19261871 PMC6666208

[ref32] Valdivia L. E.; Young R. M.; Hawkins T. A.; Stickney H. L.; Cavodeassi F.; Schwarz Q.; Pullin L. M.; Villegas R.; Moro E.; Argenton F.; Allende M. L.; Wilson S. W. Lef1-dependent Wnt/β-catenin signalling drives the proliferative engine that maintains tissue homeostasis during lateral line development. Development 2011, 138, 3931–3941. 10.1242/dev.062695. 21862557 PMC3160090

[ref33] Aleström P.; D’Angelo L.; Midtlyng P. J.; Schorderet D. F.; Schulte-Merker S.; Sohm F.; Warner S. Zebrafish: Housing and husbandry recommendations. Lab. Anim. 2020, 54, 213–224. 10.1177/0023677219869037. 31510859 PMC7301644

[ref34] Westerfield M. The Zebrafish Book: A Guide for the Laboratory Use of Zebrafish (Danio rerio), 4th ed.; University of Oregon Press: Eugene, 2000.

[ref35] Schuster K.; Ghysen A. Labeling hair cells and afferent neurons in the posterior lateral-line system of zebrafish. Cold Spring Harbor Protoc. 2013, 2013, 1172–1174. 10.1101/pdb.prot079467. 24298034

[ref36] Bátora D.; Zsigmond Á.; Lőrincz I. Z.; Szegvári G.; Varga M.; Málnási-Csizmadia A. Subcellular Dissection of a Simple Neural Circuit: Functional Domains of the Mauthner-Cell During Habituation. Front. Neural Circuits 2021, 15, 648487 10.3389/fncir.2021.648487. 33828462 PMC8019725

[ref37] Lin L.-Y.; Yeh Y.-H.; Hung G.-Y.; Lin C.-H.; Hwang P.-P.; Horng J.-L. Role of Calcium-Sensing Receptor in Mechanotransducer-Channel-Mediated Ca2+ Influx in Hair Cells of Zebrafish Larvae. Front. Physiol. 2018, 9, 649 10.3389/fphys.2018.00649. 29899708 PMC5988855

[ref38] Jain R. A.; Wolman M. A.; Marsden K. C.; Nelson J. C.; Shoenhard H.; Echeverry F. A.; Szi C.; Bell H.; Skinner J.; Cobbs E. N.; Sawada K.; Zamora A. D.; Pereda A. E.; Granato M. A Forward Genetic Screen in Zebrafish Identifies the G-Protein-Coupled Receptor CaSR as a Modulator of Sensorimotor Decision Making. Curr. Biol. 2018, 28, 1357–1369.e5. 10.1016/j.cub.2018.03.025. 29681477 PMC5940496

[ref39] Demarchi M. S.; Karenovics W.; Bédat B.; De Vito C.; Triponez F. Autofluorescence pattern of parathyroid adenomas. BJS Open 2021, 5, zraa047 10.1093/bjsopen/zraa047. 33609395 PMC7893478

[ref40] Widler L.; Altmann E.; Beerli R.; Breitenstein W.; Bouhelal R.; Buhl T.; Gamse R.; Gerspacher M.; Halleux C.; John M. R.; Lehmann H.; Kalb O.; Kneissel M.; Missbach M.; Müller I. R.; Reidemeister S.; Renaud J.; Taillardat A.; Tommasi R.; Weiler S.; Wolf R. M.; Seuwen K. 1-Alkyl-4-phenyl-6-alkoxy-1H-quinazolin-2-ones: a novel series of potent calcium-sensing receptor antagonists. J. Med. Chem. 2010, 53, 2250–2263. 10.1021/jm901811v. 20158186

[ref41] Miyazaki H.; Ikeda Y.; Sakurai O.; Miyake T.; Tsubota R.; Okabe J.; Kuroda M.; Hisada Y.; Yanagida T.; Yoneda H.; Tsukumo Y.; Tokunaga S.; Kawata T.; Ohashi R.; Fukuda H.; Kojima K.; Kannami A.; Kifuji T.; Sato N.; Idei A.; Iguchi T.; Sakairi T.; Moritani Y. Discovery of evocalcet, a next-generation calcium-sensing receptor agonist for the treatment of hyperparathyroidism. Bioorg. Med. Chem. Lett. 2018, 28, 2055–2060. 10.1016/j.bmcl.2018.04.055. 29724589

[ref42] Fanaropoulou N. M.; Chorti A.; Markakis M.; Papaioannou M.; Michalopoulos A.; Papavramidis T. The use of Indocyanine green in endocrine surgery of the neck: A systematic review. Medicine 2019, 98, e14765 10.1097/MD.0000000000014765. 30855479 PMC6417629

[ref43] Eatz T. A.; Eichberg D. G.; Lu V. M.; Di L.; Komotar R. J.; Ivan M. E. Intraoperative 5-ALA fluorescence-guided resection of high-grade glioma leads to greater extent of resection with better outcomes: a systematic review. J. Neuro-Oncol. 2022, 156, 233–256. 10.1007/s11060-021-03901-9. 34989964

[ref44] Kitagawa Y.; Tanaka S.; Kuriki Y.; Yamamoto K.; Ogasawara A.; Nejo T.; Matsuura R.; Koike T.; Hana T.; Takahashi S.; Nomura M.; Takayanagi S.; Mukasa A.; Kamiya M.; Urano Y.; Saito N. Spray Fluorescent Probes for Fluorescence-Guided Neurosurgery. Front. Oncol. 2019, 9, 727 10.3389/fonc.2019.00727. 31448231 PMC6691768

[ref45] Urano Y.; Sakabe M.; Kosaka N.; Ogawa M.; Mitsunaga M.; Asanuma D.; Kamiya M.; Young M. R.; Nagano T.; Choyke P. L.; Kobayashi H. Rapid cancer detection by topically spraying a γ-glutamyltranspeptidase-activated fluorescent probe. Sci. Transl. Med. 2011, 3, 110ra119 10.1126/scitranslmed.3002823. PMC7451965 22116934

[ref46] Li X.; Wu P.; Cao W.; Xiong H. Development of pH-activatable fluorescent probes for rapid visualization of metastatic tumours and fluorescence-guided surgery via topical spraying. Chem. Commun. 2021, 57, 10636–10639. 10.1039/D1CC04408G. 34581325

[ref47] Widen J. C.; Tholen M.; Yim J. J.; Antaris A.; Casey K. M.; Rogalla S.; Klaassen A.; Sorger J.; Bogyo M. AND-gate contrast agents for enhanced fluorescence-guided surgery. Nat. Biomed. Eng. 2021, 5, 264–277. 10.1038/s41551-020-00616-6. 32989286 PMC7969380

[ref48] Rao S. S.; Rao H.; Moinuddin Z.; Rozario A. P.; Augustine T. Preservation of parathyroid glands during thyroid and neck surgery. Front. Endocrinol. 2023, 14, 1173950 10.3389/fendo.2023.1173950. PMC10266226 37324265

[ref49] Spartalis E.; Ntokos G.; Georgiou K.; Zografos G.; Tsourouflis G.; Dimitroulis D.; Nikiteas N. I. Intraoperative Indocyanine Green (ICG) Angiography for the Identification of the Parathyroid Glands: Current Evidence and Future Perspectives. In Vivo 2020, 34, 23–32. 10.21873/invivo.11741. 31882459 PMC6984100

[ref50] Moreno-Llorente P.; García-González G.; Pascua-Solé M.; García-Barrasa A.; Videla S.; Muñoz-de-Nova J. L. Indocyanine green angiography-guided thyroidectomy versus conventional thyroidectomy for preserving parathyroid function: study protocol for a randomized single-blind controlled trial. Front. Endocrinol. 2023, 14, 1193900 10.3389/fendo.2023.1193900. PMC10200987 37223015

[ref51] Hyun H.; Park M. H.; Owens E. A.; Wada H.; Henary M.; Handgraaf H. J. M.; Vahrmeijer A. L.; Frangioni J. V.; Choi H. S. Structure-inherent targeting of near-infrared fluorophores for parathyroid and thyroid gland imaging. Nat. Med. 2015, 21, 192–197. 10.1038/nm.3728. 25559343 PMC4319985

[ref52] Wang B.; Zhu C.-R.; Liu H.; Yao X.-M.; Wu J. The Accuracy of Near Infrared Autofluorescence in Identifying Parathyroid Gland During Thyroid and Parathyroid Surgery: A Meta-Analysis. Front. Endocrinol. 2021, 12, 701253 10.3389/fendo.2021.701253. PMC8255791 34234746

